# Impact of Structural Changes on Energy Transfer in the Anion-Engineered Re^3+^:Y_2_O_3_ Through Low-Temperature Synthesis Approach

**DOI:** 10.1021/acs.jpcc.3c07132

**Published:** 2024-02-03

**Authors:** Maharram Jabrayilov, Kelly E. Cohen, Cameron L. Roman, James A. Dorman

**Affiliations:** Cain Department of Chemical Engineering, Louisiana State University, Baton Rouge, Louisiana 70803, United States

## Abstract

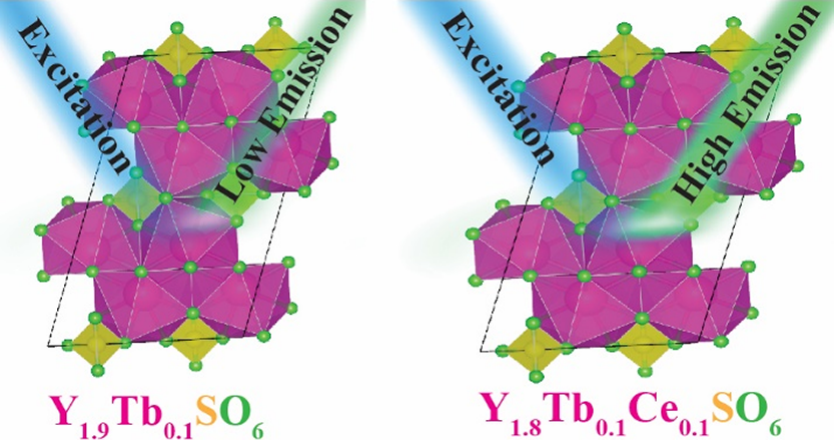

Anion engineering has proven to be an effective strategy to tailor the physical and chemical properties of metal oxides by modifying their existing crystal structures. In this work, a low-temperature synthesis for rare earth (RE)-doped Y_2_O_2_SO_4_ and Y_2_O_2_S was developed via annealing of Y(OH)_3_ intermediates in the presence of elemental sulfur in a sealed tube, followed by a controlled reduction step. The crystal structure patterns (X-ray diffraction) and optical spectra (UV-IR) of Y_2_O_2_SO_4_, Y_2_O_2_S, and crystalline Y_2_O_3_ were collected throughout the treatment steps to correlate the structural transformations (via thermogravimetric analysis) with the optical properties. Local and long-range crystallinities were characterized by using X-ray and optical spectroscopy approaches. Systematic shifts in the Eu^3+^ excitation and emission peaks were observed as a function of SO_4_^2–^ and S^2–^ concentrations resulting from a crystal evolution from cubic (Y_2_O_3_) to trigonal (Y_2_O_2_S) and monoclinic (Y_2_O_2_SO_4_), which can modify the local hybridization of sensitizer dopants (i.e., Ce^3+^). Ultimately, Tb^3+^ and Tb^3+^/Ce^3+^ doping was employed in these hosts (Y_2_O_2_SO_4,_ Y_2_O_2_S, and Y_2_O_3_) to understand energy transfer between sensitizer and activator ions, which showed significant enhancement for the monoclinic sulfate structure.

## 1 Introduction

White light-emitting diodes (w-LEDs) have substantially replaced conventional light sources (incandescent and fluorescent lamps) owing to their outstanding features such as low energy consumption,^1^ high brightness,^2^ high luminous efficiency,^3^ and long operational lifetimes.^4^ Rare earths (RE^3+^)-doped phosphors for w-LEDs have garnered considerable attention due to their widespread applications in the field of lighting.^5,6^ Among many RE-doped phosphors, Tb^3+^ doped metal oxides are well-known for obtaining green emission.^7−9^ However, Tb^3+^-doped materials suffer from a lower luminescence due to the weak and narrow absorption cross-section (σ_abs_∼ 10^–20^ cm^2^) originating from spin-forbidden electric dipole transitions between 4*f* energy levels of Tb^3+^.^10,11^ To overcome this issue, codoping of Tb^3+^ ions with Ce^3+^ sensitizers has attracted significant attention due to their high absorption cross-section (σ_abs_∼ 10^–19^ cm^2^) in the UV region stemming from the allowed 4*f*-5*d* transition, shorter luminescence lifetime, and overlapping 5*d* energy states of Ce^3+^ ions with Tb^3+ 5^D_*J*_ excited states.^12−15^ For instance, GdPO_4_:Ce^3+^/Tb^3+^ nanorods have been reported to be efficient hosts for the energy transfer from Ce^3+^ to Tb^3+^ ions with 28% quantum efficiency.^16^ The study revealed that the energy transfer depends upon the oxidation state of Ce ions in the host since dispersing codoped (GdPO_4_:Ce^3+^/Tb^3+^) sample into KMnO_4_ (oxidizing agent) solution resulted in significant luminescence quenching due to a change in the oxidation state of Ce from Ce^3+^ to Ce^4+^. Alternatively, core–shell Y_2_Sn_2_O_7_:Tb^3+^/Ce^3+^@SiO_2_ has been successfully synthesized to enhance poor energy transfer from Ce^3+^ to Tb^3+^ which stems from *D*_3d_ centrosymmetric lanthanide sites in Y_2_Sn_2_O_7_ and vibrational relaxation of surface hydroxyl groups on the nanoparticles.^17^ An improvement in the energy transfer from Ce^3+^ to Tb^3+^ is primarily associated with the migration of Ce^3+^ and Tb^3+^ ions from centrosymmetric lanthanide sites to the interface between silica and core Y_2_Sn_2_O_7_ nanoparticles. Nagashima et al. investigated the energy transfer between Ce^3+^ and Tb^3+^ in the phase-pure Ce^3+^/Tb^3+^-codoped LaLuO_3_ by selectively doping Ce^3+^/Tb^3+^ at La (A) and Lu (B) sites.^18^ The energy transfer was maximized when Tb^3+^ was doped in A sites. In contrast, another study showed that Tb^3+^/Ce^3+^ codoping in cubic phase Y_2_Zr_2_O_7_ can exhibit the luminescence quenching instead of sensitization due to the more symmetric local environment of Ce^3+^-doped sites in the host matrix and potential oxidation to Ce^4+^.^19^ Similar phenomena have been observed for Bi^3+^-Eu^3+^ ion pairs with the transition efficiency dependent on the local coordination environment, the bond distance, and the lattice symmetry.^20−22^

Over the past decade, RE-doped oxysulfates and oxysulfides have been more effective host matrices than oxides for enhancing luminescent properties.^23,24^ Various high-temperature synthesis methods have been developed to prepare RE oxysulfates and oxysulfides. For instance, Eu^3+^-doped Y_2_O_2_SO_4_ nanoparticles have been synthesized through electrospinning and combustion at 1000 °C with sulfur powder in the air.^25^ Y_2_O_2_SO_4_:Eu^3+^ was also synthesized successfully at 800 °C via a coprecipitation method employing ammonium sulfate ((NH_4_)_2_SO_4_) as a sulfur source.^26^ The charge transfer peak in the excitation and ^5^D_0_ → ^7^F_2_ transition in the emission spectrum of Eu^3+^ shifted to higher wavelengths (617–618 nm) compared with Y_2_O_3_:Eu^3+^. Low-temperature approaches for Y_2_O_2_SO_4_:Eu^3+^ have been reported^27,28^; however, the pure phase Y_2_O_2_SO_4_ is only obtained for temperatures above 950 °C. Similarly, hexagonal (trigonal) phase Yb^3+^/Er^3+^ doped Y_2_O_2_S was obtained through solid-state flux fusion with sulfur powder at 1150 °C in 1 h with internal quantum yields of green emission and infrared emission of 67 and 97%, respectively.^29^ The incorporation of S^2–^ redshifts the charge transfer band (CTB) to approximately 325 nm allowing for lower energy excitation sources.^30^ The need for high-temperature postprocessing for sulfur incorporation, release of hazardous gases, and utilization of toxic precursors (such as CS_2_) can limit the application of these materials. Thus, it is critical to develop new synthesis routes to fabricate those materials.

This study employed a low-temperature two-step facile hydrothermal/combustion process with sulfur powder to synthesize Y_2_O_2_SO_4_:RE^3+^ (Eu^3+^, Tb^3+^, Tb^3+^/Ce^3+^). Furthermore, the as-synthesized Y_2_O_2_SO_4_:RE^3+^ was converted to Y_2_O_2_S:RE^3+^ by annealing at 600 °C under a N_2_ flow, limiting the vaporization of the chalcogen. X-ray diffraction (XRD) patterns and Fourier transform infrared (FTIR) measurements have clearly confirmed the formation of crystalline nanostructures. The Eu^3+^ probe was used to elucidate the structure–property relationships in three different hosts (Y_2_O_2_SO_4_, Y_2_O_2_S, and Y_2_O_3_). Moreover, thermogravimetric analysis/differential scanning calorimetry (TGA/DSC) was employed to study the chemical transformations of as-synthesized Y_2_O_2_SO_4_ nanostructures. It showed the formation of the trigonal phase Y_2_O_2_S as the temperature reached 600 °C and further decomposition to Y_2_O_3_ at 600–800 °C. Finally, Tb^3+^/Ce^3+^ codoping was used to investigate the impact of SO_4_^2–^ and S^2–^ on the energy transfer from Ce^3+^ to Tb^3+^ ions. The photoluminescence emission (PL) and lifetime measurements show that the monoclinic sulfate host results in strong coupling of the Tb^3+^/Ce^3+^ energy levels due to modification of the 5*d* energy level and reduced local symmetries.

## 2 Experimental Section

### 2.1 Synthesis

**Figure 1:**
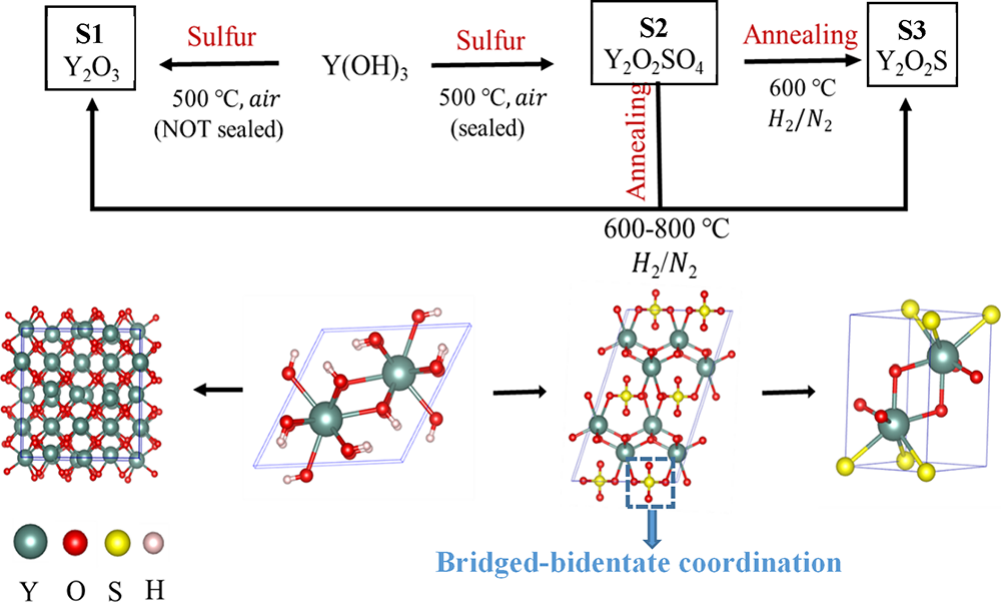
Reaction routes and crystal structures of RE-doped Y_2_O_3_ (**S1**), intermediate Y(OH)_3_, Y_2_O_2_SO_4_ (**S2**), and Y_2_O_2_S (**S3**). Crystal structures were plotted with VESTA software.^33^

Y_2_O_3_:RE^3+^ (RE = Tb, Ce, Eu) was synthesized by a modified hydrothermal method previously reported^31,32^ and highlighted in Figure 1.^33^ For instance, for the synthesis of Y_2_O_3_:Tb^3+^ (5 mol %), Ce^3+^ (5 mol %), 5.4 mmol of yttrium(III) nitrate hexahydrate (Y(NO_3_)_3_·6H_2_O, Thermo scientific, 99.9%), 0.3 mmol of terbium(III) nitrate hydrate (Tb(NO_3_)_3_·H_2_O, Alfa Aesar, 99.99%), and 0.3 mmol of cerium(III) nitrate hexahydrate (Ce(NO_3_)_3_·6H_2_O, Strem Chemicals, 99.9%) were dissolved in 12 mL of deionized water. Next, 42 mL 0.2 M NaOH (VWR Chemicals, ACS grade) was added to the solution dropwise and magnetically stirred for 2 h. After that, a milky solution was transferred to a Teflon liner autoclave and was heated up to 160 °C overnight. Then, the sample was washed with deionized water several times and dried at 100 °C overnight. This hydrothermally synthesized intermediate and sulfur were then reacted in air without sealing at 500 °C for 4 h with a 10 °C/min ramp rate in order to obtain crystalline Y_2_O_3_:Tb^3+^, Ce^3+^. Y_2_O_2_SO_4_:RE^3+^ was obtained by mixing the hydrothermally synthesized intermediate (Y(OH)_3_) and sulfur powder. For example, 200 mg of intermediate was mixed with 200 mg of excess sulfur powder (Alfa Aesar, 99.5%) and transferred into a quartz tube (20 cm long, 1 mm diameter, and 2 mm thick). Both ends of the quartz tube were sealed with a propane torch to avoid sulfur evaporation in the furnace. The sealed tube was annealed at 500 °C for 4 h with a 10 °C/min ramp rate in a box furnace. The obtained sample was washed with deionized water and toluene several times and dried at 130 °C. The obtained oxysulfate samples were further reduced to Y_2_O_2_S with 5% H_2_/95% N_2_ flow. Y_2_O_2_SO_4_ samples with various dopants were placed in a ceramic crucible and transferred to the tube furnace. Before starting the reduction, the tube furnace was purged with 5% H_2_/95% N_2_ for 30 min and ramped to 600 °C at 10 °C/min and the dwell time was 2 h.

### 2.2 Characterization

The crystal structures were obtained by using powder XRD with a PANalytical XRD operating at 45 kV and 40 mA and using a Cu Kα (λ = 1.54 Å) radiation source from 5 to 70° 2θ scan range with a step size of 0.01°. Rietveld refinement was performed on the observed XRD patterns using the General Structure Analysis System II (GSAS II) software.^34^ The following parameters were refined: histogram scale factor, displacement, lattice parameters, microstrain, crystallite size, instrument parameters, background, atomic coordinates, and thermal parameters. An Edinburgh FLS1000 PL spectrometer equipped with a PMT detector and a 450 W ozone-free xenon arc lamp as a light source was employed to perform the photoluminescence and lifetime measurements. A quartz spectrophotometer cell (Starna Cells, Inc.) was used to place the powder samples to obtain the room-temperature emission and excitation spectra. The PL measurements were collected with a bandwidth of 1.5 nm, a dwell time of 0.4 s, and a step size of 1 nm in the ranges of 200–400 nm (excitation) and 470–700 nm (emission), respectively. A 455 nm filter was employed to reduce the fluorescence from the lamp for emission measurements. The lifetime measurements were obtained at 77 K with a microsecond flash lamp (frequency: 25 kHz, 1–2 μs pulse) over a range of 10 ms with a 2 ms delay time, resulting in a 5 μs detector response. To quantify lifetime values for all hosts, decay curves of all samples were fitted with an exponential formula:
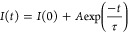
1

where *I* and *t* are the luminescence intensity and time (μs), respectively, *A* is the fitting constant, and τ is the lifetime. Attenuated total reflectance Fourier transform infrared (ATR-FTIR) measurements were carried out in a Thermo Scientific Nicolet 6700 FTIR instrument equipped with a Smart iTR sampling accessory (Thermo Scientific). A 2–3 mg sample was mixed and ground with 100 mg of KBr (EMD chemicals, ACS grade) and was placed on the crystal plate and pressed. All spectra were collected in a scan range from 1400 to 500 cm^–1^ with a 4 cm^–1^ spectral resolution by averaging 128 scans. Inductively coupled plasma-optical emission spectroscopy (ICP-OES) measurements were performed using a PerkinElmer Optima 8000. All samples were prepared by digesting 20 mg of the Y_2_O_3_:Tb^3+^ (5 mol %), Ce^3+^ (5 mol %), Y_2_O_2_S:Tb^3+^ (5 mol %), Ce^3+^ (5 mol %), Y_2_O_2_SO_4_:Tb^3+^ (5 mol %), and Ce^3+^ (5 mol %) in an aqueous HNO_3_ (MiliporeSigma, 65%) and HCl (VWR BDH Chemicals, 38%). For the measurement, the digested samples were diluted to 12–15 ppm by using 2% HNO_3_. Meanwhile, Y, Tb, Ce, and S standards (1000 ppm, Inorganic Ventures) were diluted to 1, 5, 10, and 20 ppm with 2% HNO_3_ for the calibration. According to the ICP-OES measurements, the actual concentrations of Tb^3+^ and Ce^3+^ were 3 mol % for this sample, as shown in Table S2. TGA/DSC was performed using a TA SDT Q600. About 18 mg of Y_2_O_2_SO_4_ was loaded to a sample holder and ramped at 5 °C/min to 800 °C with a final dwell time of 2 h. For reducing these samples, a 5% H_2_/95% N_2_ flow was used.

## 3 Results and Discussion

**Figure 2:**
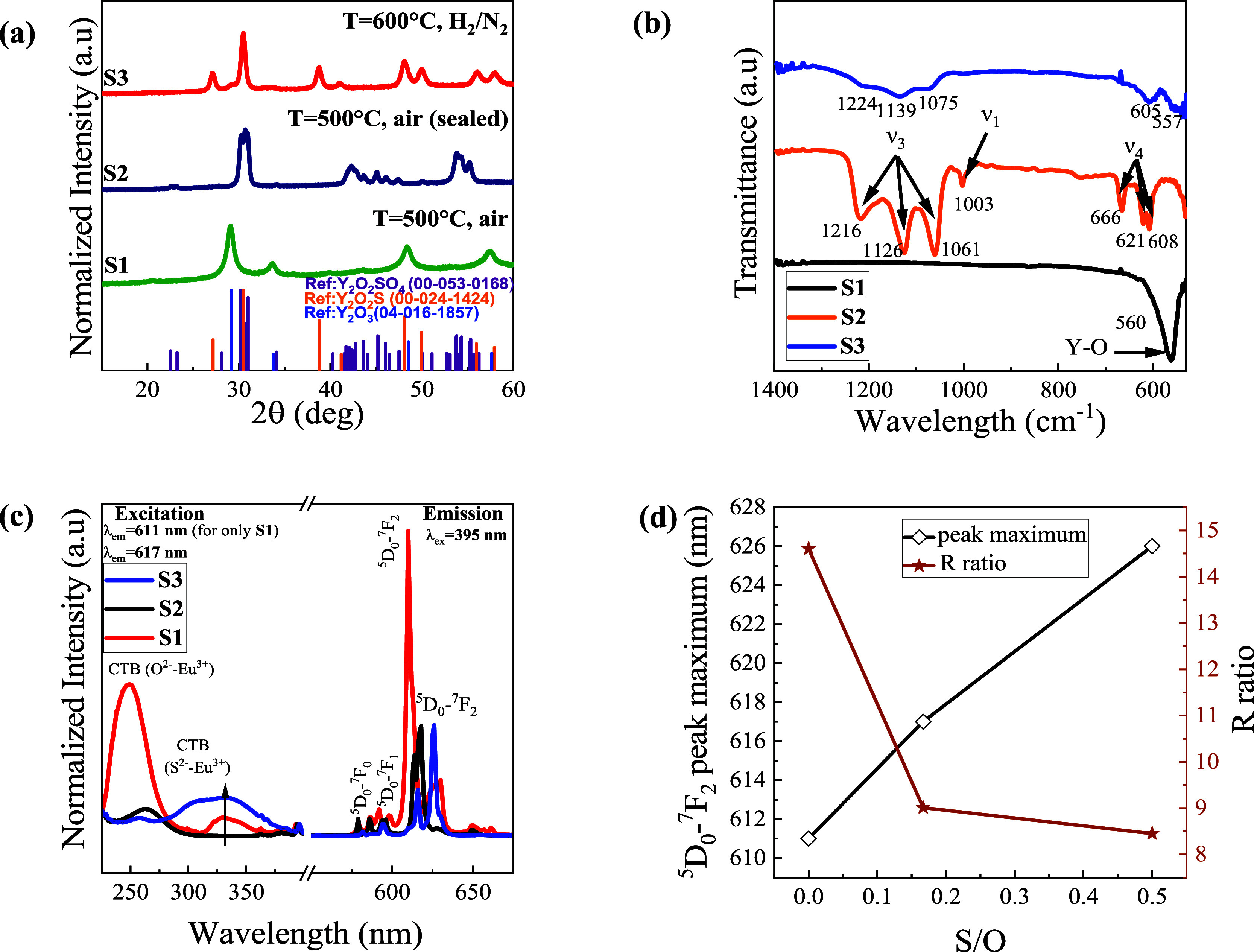
(a) XRD patterns of 5 mol % Eu^3+^-doped hydrothermally synthesized precursor/sulfur at 500 °C with air (**S1**), 5 mol % Eu^3+^ doped hydrothermally synthesized precursor/sulfur at 500 °C with air in a sealed quartz tube (**S2**) and the reduction of as-synthesized S@ at 600 °C with 5% H_2_/N_2_ flow (**S3**). (b) FTIR spectra of samples **S1**, **S2**, and **S3**, which shows the IR active vibrations for the different hosts. (c) Excitation spectra of **S1**, **S2**, and **S3** at 611 nm (only for S1) and 617 nm emission and emission spectra of **S1**, **S2**, and **S3** at 395 nm excitation. (d) Relations of the wavelength of the electric dipole transition (^5^D_0_→ ^7^F_2_) at the peak maximum and the asymmetric ratios (*R*) as a function of the S/O molar ratios.

Y_2_O_3_, Y_2_O_2_SO_4_, and Y_2_O_2_S were synthesized and denoted as **S1**, **S2**, and **S3**, respectively. The XRD patterns of the samples are shown in Figure 2a. For clarity, the XRD pattern of the **S2** was shown with a magnified view, specifically highlighting the angle range between 28.5 and 32.5°, in the Supporting Information (Figure S1). All samples were obtained from the same hydrothermal synthesis method^32,35^ and crystallite sizes, based on the Scherrer equation, for **S1**, **S2**, and **S3** were found to be roughly 20.62, 22.59, and 21.86 nm, respectively. This suggests that the chemical transformation does not necessarily impact the crystallite sizes. Sample **S1** matched well with the cubic phase Y_2_O_3_ with space group *Ia*3̅ (JCPDS card No.04-016-1857).^36^ Since the sulfur evaporates at low temperatures (around 444 °C) at standard atmospheric pressure,^37^ the air flow in the tube furnace is believed to remove sulfur vapors quickly, resulting in precursor dehydration to form Y_2_O_3_. Nevertheless, when the precursor and sulfur mixture were reacted in the sealed quartz tube with air, a new diffraction pattern associated with **S2** was obtained. The XRD pattern can be indexed to the monoclinic phase of Y_2_O_2_SO_4_ with space group *C*2/*c* (JCPDS card No.00-053-0168) confirming the incorporation of sulfate (SO_4_^2–^) ions. No other impurity phases were observed. Furthermore, the reduced **S2** samples resulted in a secondary transformation in the diffraction pattern due to the formation of **S3.** This transformation is attributed to the presence of Y_2_O_2_S. Minor peaks are observed around 29.1° corresponding with Y_2_O_3_ impurities due to overreduction of the samples. The crystal structure of sample **S3** agrees well with that of the trigonal phase Y_2_O_2_S (space group: *P*3̅*m*1, JCPDS card No.00-024-1424).^38^ All three diffraction patterns and their refinements are shown in the Supporting Information (Figure S2), including the associated peak positions, to confirm the phase purity of the as-synthesized materials.Y_2_O_3_ was refined with the cubic phase Y_2_O_3_,^36^ while Y_2_O_2_S and Y_2_O_2_SO_4_ were refined from the trigonal phase Y_2_O_2_S^39^ and the monoclinic phase Eu_2_O_2_SO_4_,^40^ respectively. Atomic positions and isotropic displacement parameters for all samples (*U*_iso_) are given in the Supporting Information (Table S1). The refinement results indicate that the synthesis methods are possible low-temperature routes to obtain relatively defect-free nanostructures. The lattice parameters and statistical factors of all samples confirm the reasonable fitting of the experimental data (Table 1).

**Table 1:** Crystallographic Data and Structure Parameters from Rietveld Refinement (All Samples Doped with 5 mol % Eu^3+^)

cell parameters	Y_2_O_3_	Y_2_O_2_S	Y_2_O_2_SO_4_
space group	*Ia3̅* (cubic)	*P3̅m1* (trigonal)	*C2/c* (monoclinic)
*a* (Å)	10.671	3.781	13.308
*b* (Å)	10.671	3.781	4.142
*c* (Å)	10.671	6.598	8.051
α, β, γ (◦)	90, 90, 90	90, 90, 120	90, 107.83, 90
*V* (Å^3^)	1215.13	81.70	422.52
*R*_wp,_*R*_p_ (%)	2.96, 2.25	6.04, 4.59	3.73, 2.80
χ^2^	3.85	7.64	8.85

As further confirmation, FTIR spectroscopy was used to probe the structural and vibrational frequencies of the synthesized crystals, as shown in Figure 2b, specifically focusing on vibrations stemming from sulfur bonds. For sample **S1**, the strong absorption band located at 560 cm^–1^ is associated with Y–O lattice vibrations which confirm the formation of crystalline phase Y_2_O_3_ nanostructures.^41^ In sample **S2**, the peaks located at 1061, 1126, and 1216 cm^–1^ are attributed to asymmetric ν_3_ (SO_4_^2–^) stretches.^42^ The peak centered at around 1003 cm^–1^ is due to ν_1_ (SO_4_^2–^), and peaks at 608, 621, and 666 cm^–1^ were assigned as the ν_4_ (SO_4_^2–^).^43^ The appearance of strong triply degenerate ν_3_ and ν_4_ modes indicates that the point symmetry of the sulfate group is distorted from *T*_d_ to *C*_2ν_.^44^ Such a distortion shows that the coordination between SO_4_^2–^ and Y^3+^ is the bridged bidentate-type, in which two oxygen atoms from the sulfate group are directly coordinated with Y^3+^ and the rest of them are uncoordinated. The single vibrational mode around 1003 cm^–1^ (ν_1_) is usually IR-inactive, but it was observed in FTIR spectra due to the low symmetry in the oxysulfate.^45^ Moreover, after the annealing of Y_2_O_2_SO_4_ in the hydrogen environment, the splits around 1100 cm^–1^ became narrower and blue-shifted approximately 14 cm^–1^, and the splits between 608 and 670 cm^–1^ disappeared, resulting from the reduction of SO_4_^2–^ groups to S^2–^ ions.^26^ This is due to the trigonal crystal structure of Y_2_O_2_S where S^2–^ is directly bonding with Y^3+^ and forms a stronger bond, which increases the frequency.

To further understand the local crystal structure, the samples were doped with 5 mol % Eu^3+^ to take advantage of the crystal structure-dependent luminescence associated with the electric (∼615 nm) and magnetic dipole (590 nm) transitions.^46,47^ Low RE-dopant concentrations (up to 15 mol %) have been shown to have little to no impact on the crystal structures due to the similarities of the RE^3+^ and Y^3+^ ionic radii.^35,48^ Photoluminescent excitation and emission spectra were collected at room temperature for all three samples (Figure 2c). The sample **S1** exhibits a strong, broad excitation band with a maximum value at 248 nm owing to the CTB between O^2–^ and Eu^3+^.^49^ With the incorporation of sulfate ions, the CTB maximum redshifts to 267 nm for the **S2**. A reduction of this sample to Y_2_O_2_S:Eu^3+^ (5 mol %, **S3**) resulted in an appearance of an O^2–^-Eu^3+^ band at approximately 260 nm, with additional broadband peaks between 300 and 375 nm, indicative of a 3*p* (S^2–^) to 4*f* (Eu^3+^) CTB.^50^ The CTB redshift is attributed to the different electronegativities, specifically 3.44 (O) and 2.6 (S), resulting in weaker Eu–O bonding.^51^ For sample **S3**, the shift in excitation peak around 260 nm has been reported to be a function of both the O^2–^-Eu^3+^ hybridization and the Y_2_O_2_S adsorption edge.^52^ Furthermore, the broad CTB between 300 and 375 nm is due to S^2–^-Eu^3+^ hybridization, and is consistent with the crystal structure of Y_2_O_2_S where each Eu^3+^ is directly coordinated with three S^2–^. Jörgensen proposed an empirical formula to calculate energy of charge transfer between anion and metal ions given by
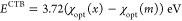
2

where χ_opt_(*x*) and χ_opt_(*m*) are the optical electronegativities of the anion (*x*) and metal (*m*).^53^ The calculated CTB energies of Y_2_O_3_:Eu^3+^, Y_2_O_2_SO_4_:Eu^3+^, and Y_2_O_2_S:Eu^3+^ were reported by Dorenbos as 5.12, 4.59, and 3.61 eV, respectively.^54^ Thus, by using an equation of λ = 1240/*E*^CTB^, the CTB wavelengths can be estimated as 242, 270, and 343 nm for Y_2_O_3_:Eu^3+^, Y_2_O_2_SO_4_:Eu^3+^, and Y_2_O_2_S:Eu^3+^, respectively, agreeing well with the above results.^55^ Moreover, the emission spectra of all three samples were obtained under 395 nm excitation. The predominant red emission was observed at 611 nm for the **S1**, and this is attributed to the electric dipole transitions ^5^D_0_ → ^7^F_2_. The electric dipole transitions redshifted to 617 and 626 nm for samples **S2** and **S3,** respectively. Figure 2d highlights the correlation between the S/O stoichiometric mole ratios and the wavelength of the electric dipole transition ^5^D_0_ → ^7^F_2_ at the peak maximum and the asymmetric ratio (*R*). The *R* value is defined as the intensity ratio of ^5^D_0_ → ^7^F_2_ to ^5^D_0_ → ^7^F_1_, which describes the electric and magnetic dipole transitions, the former of which is known to be symmetry-dependent.^56^ Larger *R* values are indicative of lower local symmetry and better luminescence. The highest *R* ratio (14.7) was observed in the Y_2_O_3_. Usually, Eu^3+^ ions occupy either C_2_ or S_6_ sites in the cubic phase Y_2_O_3_ host; the latter one has more symmetric coordination than the former sites. Here, the largest asymmetry ratio designates Eu^3+^ ions to occupy mainly C_2_ sites in the Y_2_O_3_, which agrees well with previously reported values.^57^ Nevertheless, the *R* ratios for the monoclinic Y_2_O_2_SO_4_ (8.9) and trigonal Y_2_O_2_S (8.4) are lower than the Y_2_O_3_. This is attributed to the incorporation of SO_4_^2–^ and S^2–^,which reduced the asymmetry of Eu^3+^ in the *C*_1_ and *C*_3v_ sites, respectively. To the best of our knowledge, there are no reported *R* values for the Y_2_O_2_SO_4_ host, but the *R* value for Y_2_O_2_S was found to be approximately 6.50, which is consistent with our calculated value.^58^

**Figure 3:**
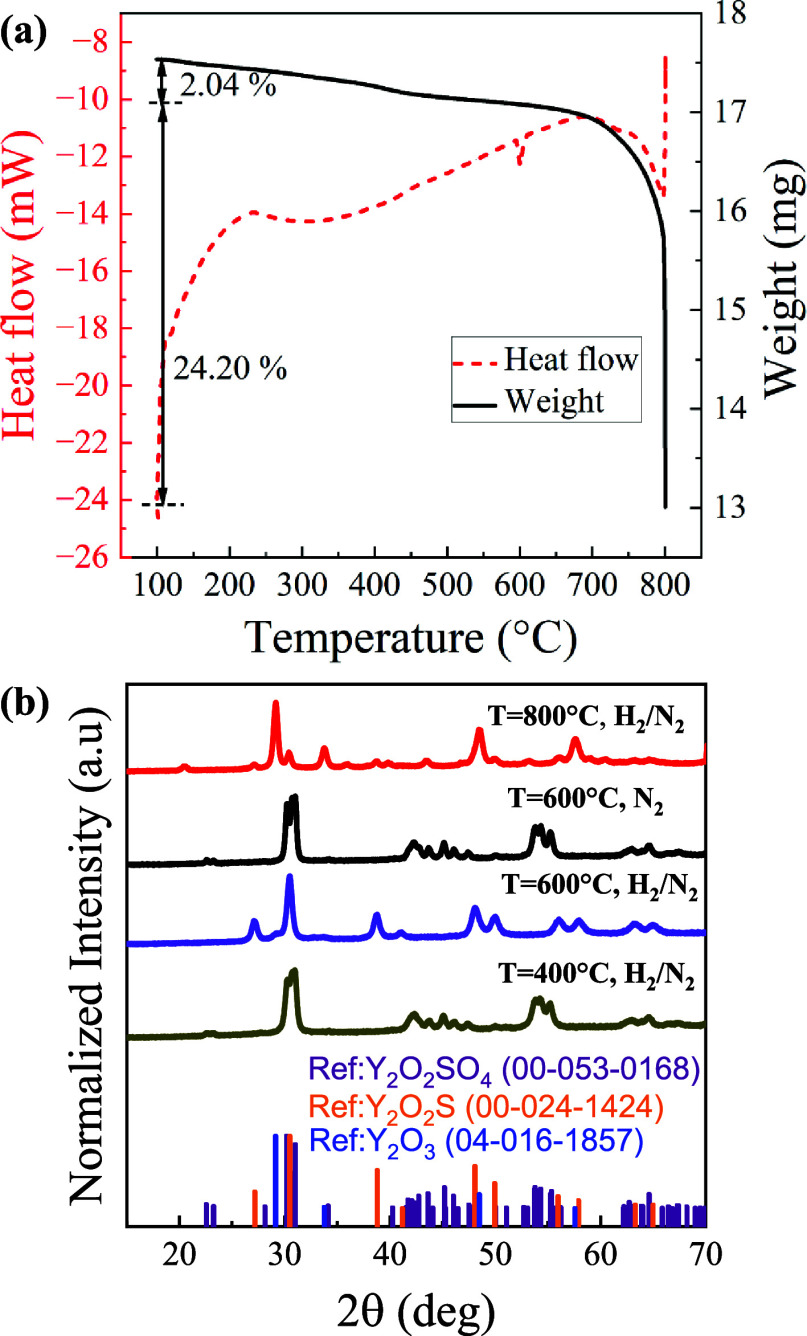
Chemical transformations of Y_2_O_2_SO_4_. (a) TGA/DSC (5% H_2_/ 95% N_2_) for the Y_2_O_2_SO_4_. (b) XRD profiles of Y_2_O_2_SO_4_ samples annealed at different temperatures and environments.

Next, to explore the Y_2_O_2_SO_4_ phase transformation in situ, the samples were annealed in an H_2_/N_2_ (5:95) gas in the TGA/DSC apparatus with a ramp rate of 5 °C/min (Figure 3a). A small (2%) reduction of mass is measured below 400 °C and is associated with the physisorbed/chemisorbed species (water and toluene) used to wash the samples. The diffraction pattern for an equivalent sample (Figure 3b) exhibits no change in structure at 400 °C confirming the loss of surface species during low-temperature treatments. Further annealing of the samples shows minimal weight loss and heat flux until 600 °C. At this temperature, a small endothermic peak is observed followed by a rapid loss in mass from 600 to 800 °C. The first reduction is due to the extraction of oxygen ions from the sulfate group resulting in the conversion of Y_2_O_2_SO_4_ to Y_2_O_2_S. This reaction can be written as
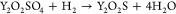
3

This agrees well with a previous experiment in which Y_2_O_2_SO_4_ samples were reduced to Y_2_O_2_S when calcined at 600 °C under H_2_/N_2_ flow (with a dwell time of 2 h), as shown in Figure 3b. The complete conversion of Y_2_O_2_SO_4_ to Y_2_O_2_S is expected to result in a 20.9% change in mass. However, the total mass loss between 600 and 800 °C was experimentally observed to be approximately 24.2%, suggesting a secondary transformation is occurring, i.e., the decomposition of Y_2_O_2_SO_4_ to Y_2_O_3_. This transformation would result in a 26.9% theoretical mass loss, indicating that in our TGA/DSC experiment, the rate of decomposition is much slower than the rate of reduction at higher temperatures (600–800 °C). This agrees well with the XRD data for 800 °C where mainly Y_2_O_3_ peaks, with an impurity Y_2_O_2_S crystal phase, were observed (Figure 3b). A reference reaction in N_2_ shows no mass or phase change up to 600 °C confirming the H_2_-induced reduction (Figure 3b).

**Figure 4:**
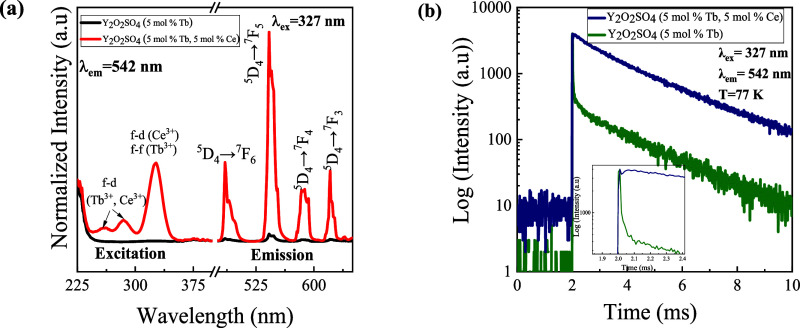
Photoluminescent properties of Y_2_O_2_SO_4_ (5 mol % Tb^3+^) and Y_2_O_2_SO_4_ (5 mol % Tb^3+^, 5 mol % Ce^3+^). (a) PL excitation spectra at an emission wavelength of 542 nm and PL emission spectra excited at 327 nm. (b) Logarithmic lifetime plots of Y_2_O_2_SO_4_ (5 mol % Tb^3+^) and Y_2_O_2_SO_4_ (5 mol % Tb^3+^, 5 mol % Ce^3+^) with an excitation wavelength of 327 nm and an emission wavelength of 542 nm at 77 K.

Next, the samples were doped with Tb^3+^ (5 mol %) and Tb^3+^/Ce^3+^ (5 mol %/5 mol %) to understand the role of the different hosts on the energy transfer and luminescence. Figure 4a shows the photoluminescence emission and excitation (PLE) and PL spectra of Y_2_O_2_SO_4_ (5 mol % Tb^3+^) and Y_2_O_2_SO_4_ (5 mol % Tb^3+^, 5 mol % Ce^3+^). The excitation spectra are normalized to the RE-O/RE-S CTB in order to eliminate the intensity variations due to sample loading. The excitation peaks of the codoped host at 258 and 285 nm are ascribed to electric dipole-allowed *f–d* transitions of the Ce^3+^ and Tb^3+^ions.^16,59^ The excitation peak above 300 nm corresponds to *f–d* transitions of the Ce^3+^ and *f–f* transitions of Tb^3+^.^60,61^ Furthermore, the emission spectra were collected at 327 nm excitation. In both samples, the characteristic Tb^3+^ emission bands at 485 (^5^D_4_ → ^7^F_6_), 542 (^5^D_4_ → ^7^F_5_), 585 (^5^D_4_ → ^7^F_4_), and 620 nm (^5^D_4_ → ^7^F_3_) were observed. However, the codoped sample has a significantly higher intensity than the singly doped sample. This is indicative of the energy transfer from Ce^3+^ to Tb^3+^. To verify this, low-temperature (77 K) lifetime measurements were performed (Figure 4b). The shape of the white line region (at 2 ms), i.e., short increase before decay onset, is a signature of energy transfer between two activator ions or between the sensitizer and activator.^62^ As can be seen from the inset of Figure 4b, the Tb^3+^ doped samples show an immediate, rapid decay commonly seen with single absorber/emitter systems. The low-temperature lifetime measurements indicate that the RE^3+^ dopant is demonstrating high efficiencies based on comparable Tb systems.^19,48^ The comparative assessment of lifetime values within codoped and singly doped samples revealed a discernible increase in the former. This observed enhancement, as demonstrated in prior investigations concerning Tb^3+/^Ce^3+^ doped systems,^17,63^ was elucidated through the conceivable occurrence of energy transfer mechanisms between the two distinct dopants due to the inherent symmetry-driven energy transfer dynamics. Another plausible explanation for the energy transfer can be the change in the phonon dispersion of the lattice with the replacement of the anion with SO_4_^2–^ which was explored in the previous Tb^3+^ doped oxyhalide systems.^64^ When Tb^3+^ and Ce^3+^ ions are in proximity, their energy states can be coupled with the phonon modes of the crystal lattice. This coupling enables the transfer of energy between dopants, modifying their excited state lifetimes.

Additionally, the lifetime measurements for the other hosts (Y_2_O_2_S and Y_2_O_3_) were recorded as shown in Figures S3a–d and S4a–d, and both hosts showed similar behavior for the doped and codoped systems (Table 2). Compared with Y_2_O_2_SO_4_, Ce^3+^/Tb^3+^-doped Y_2_O_2_S exhibited lifetime values lower than those of the Tb^3+^-doped sample, indicating no energy transfer between the dopants. In fact, luminescence is quenched when Ce^3+^ is codoped into Y_2_O_2_S due to the trigonal crystal dopant sites (*C*_3v_ symmetry) modifying the dopant crystal field splitting and limited overlap of the 5*d* level with the Tb^3+5^D_4_ → ^7^F_5_ energy levels.^65^ As a result, a reverse energy transfer from the ^5^D_4_ energy level of Tb^3+^ to the 5*d* energy level of Ce^3+^ can occur. Similar energy transfer mechanisms have been observed previously.^65−67^ At 285 nm excitation, a lifetime value for the codoped Y_2_O_3_ sample could not be calculated due to a weak emission intensity resulting from significant quenching due to parasitic surface sites.^68^ Furthermore, the 285 nm excitation of Y_2_O_3_ (5 mol % Tb^3+^) has a shorter lifetime that is due to its cubic phase crystal structure with Tb^3+^ ions occupying the high symmetry S_6_ site.^69^

**Table 2:** Calculated Lifetime Values of All Three Hosts from Exponential Fitting Function

sample	τ (μs)		τ (μs)	
	λ_ex_ = 285 nm, λ_em_ = 542 nm at 77 K		λ_ex_ = 327 nm, λ_em_ = 542 nm at 77 K	
	5 mol % Tb^3+^	5 mol % Tb^3+^	5 mol % Tb^3+^	5 mol % Tb^3+^
		5 mol % Ce^3+^		5 mol % Ce^3+^
Y_2_O_3_	385.44			
Y_2_O_2_S	704.97	343.59	829.41	784.7
Y_2_O_2_SO_4_	1012.46	1110.1	1497.27	1624.06

## 4 Conclusions

In summary, Y_2_O_2_SO_4_:RE^3+^ (Eu^3+^, Tb^3+^, Tb^3+^/Ce^3+^) has been successfully prepared via a low-temperature two-step hydrothermal/combustion method. The as-synthesized samples were further reduced to Y_2_O_2_S under an H_2_/N_2_ flow at 600 °C. XRD, FTIR, and PL measurements were used to extract the structural properties, the nature of bonding in the crystal lattices, and relationships between structural and photoluminescent properties, respectively. Rietveld refinement of the XRD data confirmed the crystallographic and structural parameters of all three hosts (Y_2_O_2_SO_4_, Y_2_O_2_S, and Y_2_O_3_). The chemical transformations under different environments have been examined by TGA/DSC measurements, and the phases obtained were confirmed by XRD. Ultimately, Tb^3+^ and Ce^3+^ doping was employed to study energy transfer mechanisms in three hosts with different crystal structures. PL spectra and lifetime measurements show that the Y_2_O_2_SO_4_ host maximizes the energy transfer from Ce^3+^ to Tb^3+^ and enhances the green emission of Tb^3+^ ions due to the structure altering both the local symmetry/environment, phonon dispersion around the RE ions and the relative position of the Ce^3+^ CTB with respect to the Tb^3+^ energy levels. These results indicate that photoluminescent properties of RE-doped oxides can be tailored via anionic substitution, which can allow for the design of novel phosphor materials with enhanced properties for the application of w-LEDs, solid-state lasers, and noncontact thermometers.

## Supporting Information Available

XRD pattern of the Y_2_O_2_SO_4_ (5 mol % Eu^3+^), Rietveld refinement fittings, atomic positions/isotropic displacement parameters from GSAS II, logarithmic lifetime plots, ICP-OES analysis results (PDF)
