# Supplementary material for: Impact of Structural Changes on Energy Transfer in the Anion-Engineered Re3+:Y2O3 Through Low-Temperature Synthesis Approach
Source: J Phys Chem C Nanomater Interfaces. 2024 Feb 3;128(6):2625–33. doi: 10.1021/acs.jpcc.3c07132 (PMC10875659; doi:10.1021/acs.jpcc.3c07132)
Supplement: Supplementary file 1 — jp3c07132_si_001.pdf [file jp3c07132_si_001.pdf]

Supporting Information:

Impact of Structural Changes on Energy Transfer in the Anion-Engineered Re^{3+}:Y_2O_3 Through Low-Temperature Synthesis Approach

Maharram Jabrayilov [a], Kelly E. Cohen [a], Cameron L. Roman [a], James A. Dorman [a],*

[a] Cain Department of Chemical Engineering, Louisiana State University, Baton Rouge, Louisiana 70803, United States

Corresponding author, jadorman@gmail.com

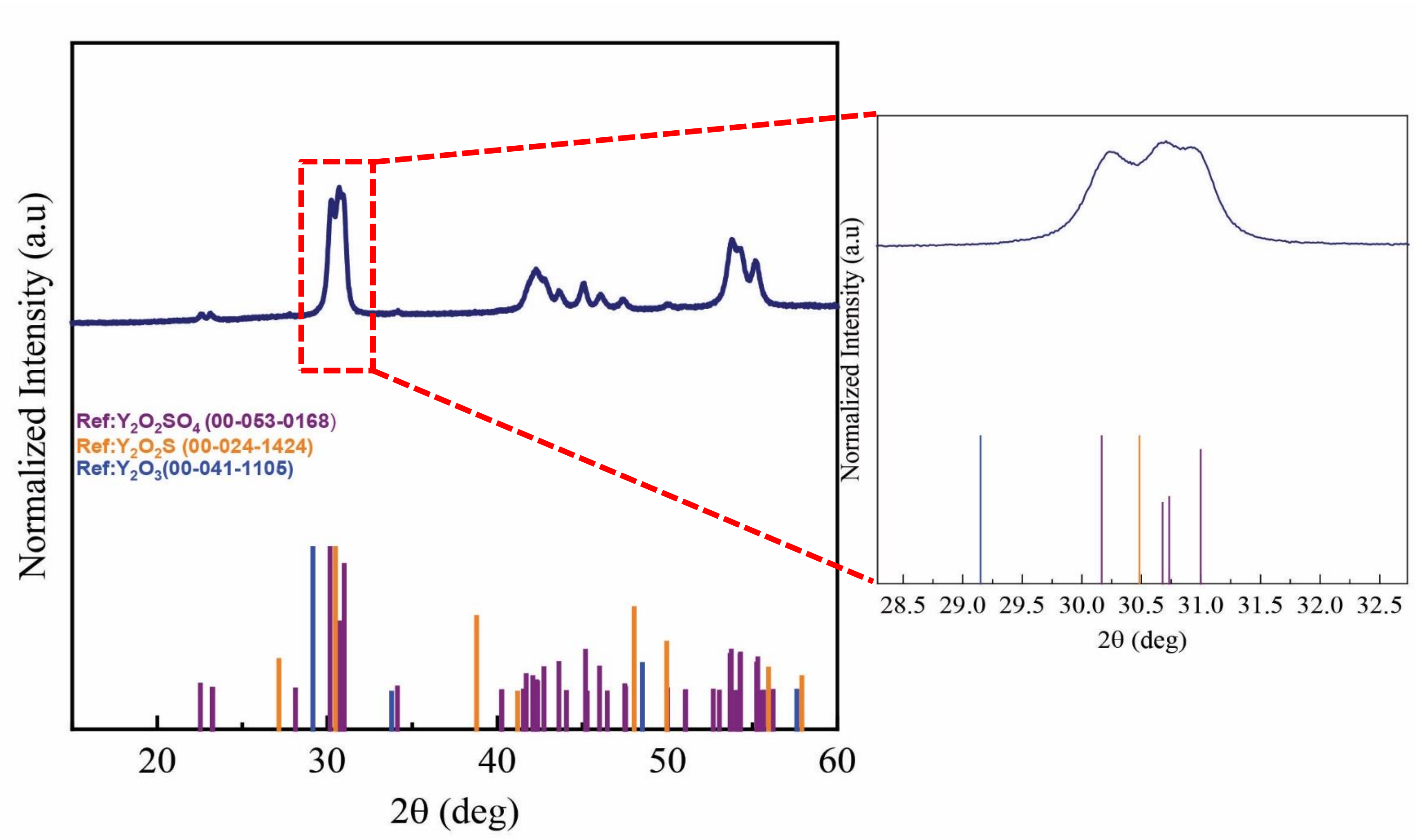

Figure S1. XRD pattern of the as-synthesized $Y_2O_2SO_4$ (5 mol % Eu^{3+}).

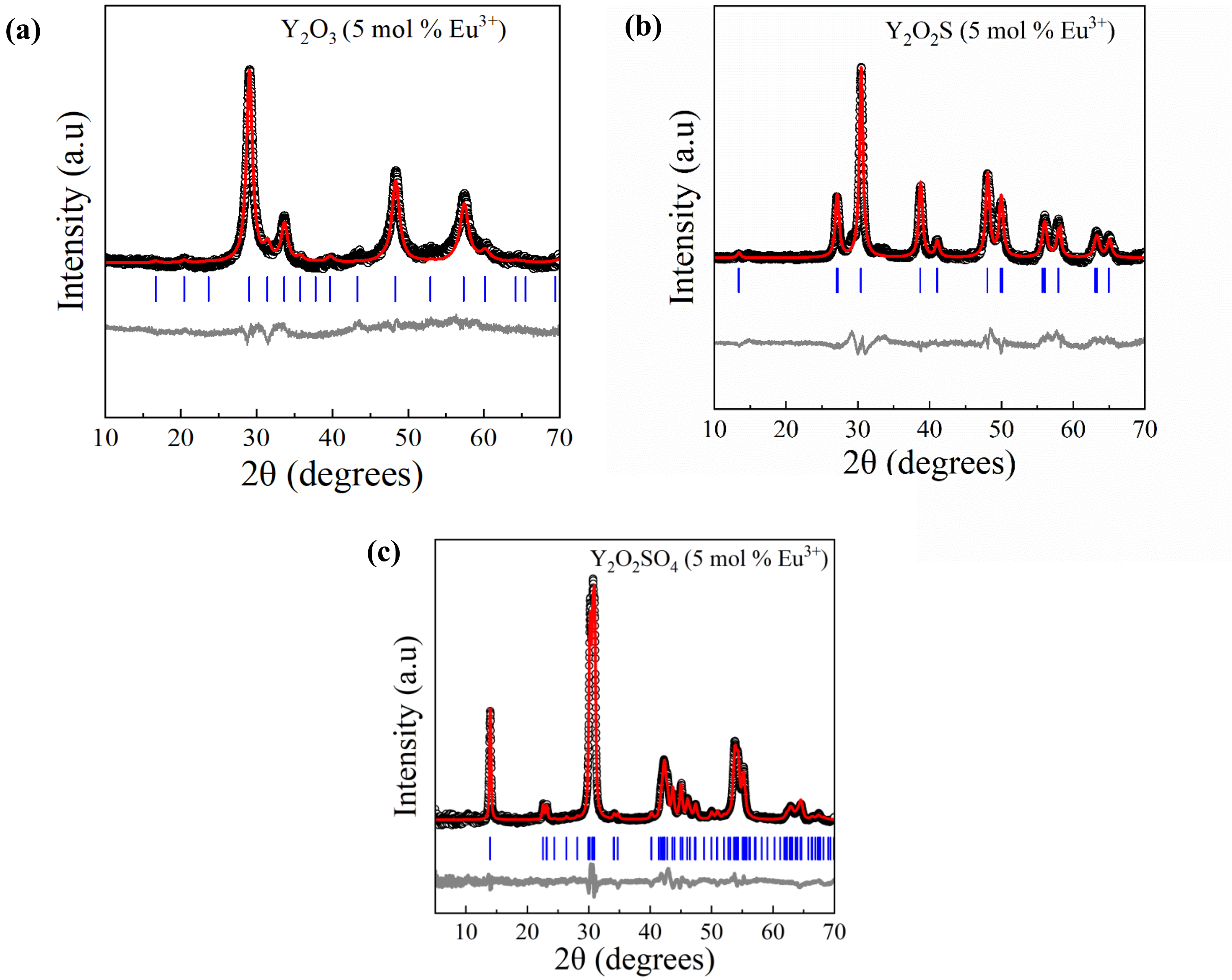

Figure S2. Rietveld Refinement with GSAS II: (a) Y_2O_3 (5 mol% Eu^{3+}), (b) Y_2O_2S (5 mol% Eu^{3+}), (c) $Y_2O_2SO_4$ (5 mol% Eu^{3+}). Black circles are experimental data, red lines are fit, grey lines are difference, and blue lines are calculated peak positions.

Table S1. Atomic positions and isotropic displacement parameters (U_{iso}) obtained through Rietveld Refinement of samples.

Atoms	x	y	z	U_{iso}
**Y_2O_3 (5 mol% Eu^{3+})**				
**Y1**	0.97906	0.00000	0.25000	0.01704
**Y2**	0.25000	0.25000	0.25000	0.03262
**O1**	0.40660	0.16606	0.40912	0.00407
**Y_2O_2S (5 mol% Eu^{3+})**				
**Y1**	0.33333	0.66666	0.36491	0.00291
**O1**	0.33333	0.66666	0.66642	0.00000
**S1**	0.00000	0.00000	0.00000	0.00000
**$Y_2O_2SO_4$ (5 mol% Eu^{3+})**				
**Y1**	0.16789	0.50665	0.08229	0.01315
**O1**	0.24857	1.06551	0.15493	0.01500
**O2**	0.99956	0.24011	0.11388	0.00697
**O3**	0.10925	0.88162	0.33587	0.02484
**S1**	0.00000	0.03456	0.25000	0.01844

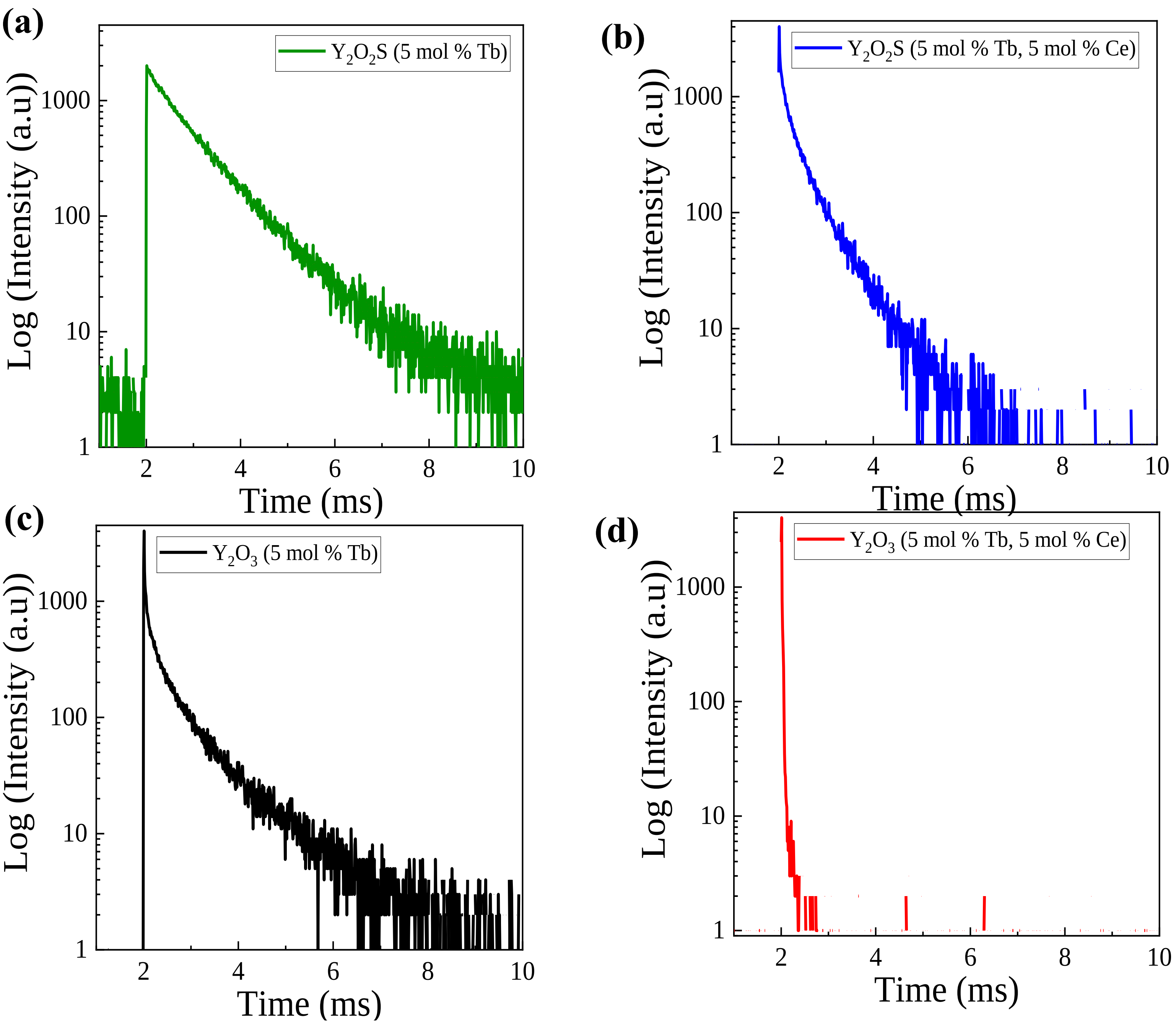

Figure S3. Logarithmic lifetime plots at λ_{ex} = 285 nm, λ_{em} = 542 nm at **77 K**. (a)Y_2O_2S: 5 mol % Tb, (b) Y_2O_2S: 5 mol % Tb, 5 mol % Ce, (c) Y_2O_3:5 mol % Tb, and (d)Y_2O_3:5 mol % Tb, 5 mol % Ce.

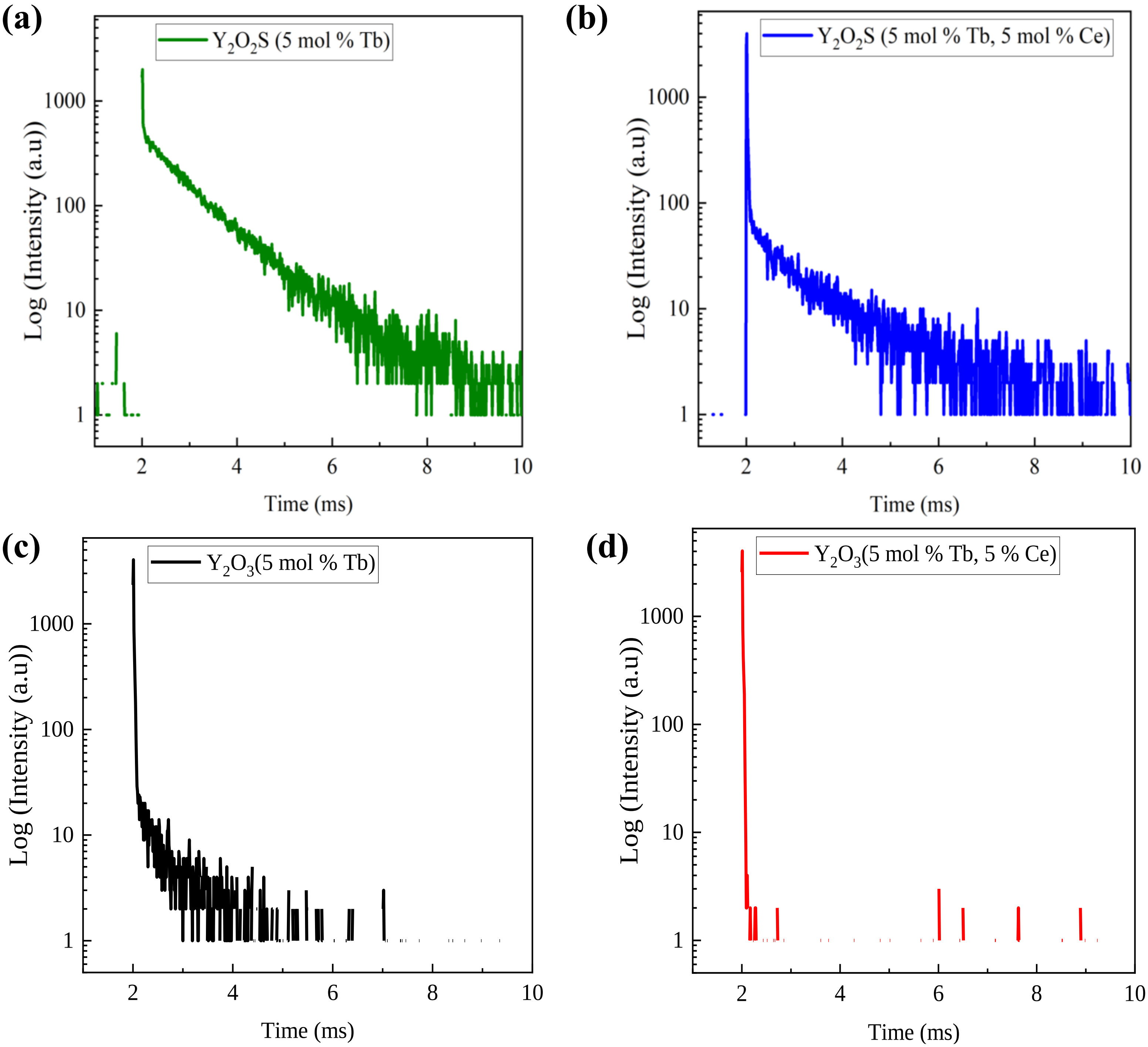

Figure S4. Logarithmic lifetime plots at $\lambda_{ex} = 327$ nm, $\lambda_{em} = 542$ nm at **77 K**. (a)Y_2O_2S: 5 mol % Tb, (b) Y_2O_2S: 5 mol % Tb, 5 mol % Ce, (c) Y_2O_3:5 mol % Tb, and (d)Y_2O_3:5 mol % Tb, 5 mol % Ce.

Table S2. ICP-OES data of the Y_2O_3 (5 mol % Tb) and Y_2O_3 (5 mol % Tb, 5 mol % Ce), the Y_2O_2S (5 mol % Tb) and Y_2O_2S (5 mol % Tb, 5 mol % Ce) and the $Y_2O_2SO_4$ (5 mol % Tb) and $Y_2O_2SO_4$ (5 mol % Tb, 5 mol % Ce).

Samples	ICP-OES (mg/L)	ICP-OES (mmol/L)	ICP-OES (mol %)*
**Y_2O_3 (5 mol% Tb, 5 mol% Ce)**	Y (20.970)<br>Tb (1.189)<br>Ce (1.151)	Y (0.236)<br>Tb (0.007)<br>Ce (0.008)	Y (93.76)<br>Tb (2.98)<br>Ce (3.26)
**Y_2O_2S (5 mol % Tb, 5 mol % Ce)**	Y (25.698)<br>Tb (1.409)<br>Ce (1.380)<br>S (4.52)	Y (0.289)<br>Tb (0.008)<br>Ce (0.009)<br>S (0.141)	Y (64.65)<br>Tb (1.79)<br>Ce (2.01)<br>S (31.54)
**$Y_2O_2SO_4$ (5 mol % Tb, 5 mol % Ce)**	Y (26.719)<br>Tb (1.923)<br>Ce (1.981)<br>S (4.978)	Y (0.303)<br>Tb (0.012)<br>Ce (0.014)<br>S (0.156)	Y (62.47)<br>Tb (2.47)<br>Ce (2.89)<br>S (32.16)

** For calculation of molar percentages of elements, we assumed that there were no any oxygen vacancies present in the samples.*
